# Enhanced Physico-Mechanical Properties of Sericin–PVA Composite Films with a Potential Antibacterial and Controlled Drug Release Features for Wound Dressing

**DOI:** 10.3390/ijms27125216

**Published:** 2026-06-09

**Authors:** Kanono Comet Manesa, Simiso Dube, Mathew Muzi Nindi

**Affiliations:** Department of Chemistry, Florida Science Campus, University of South Africa, Roodepoort 1709, South Africa; dubes@unisa.ac.za (S.D.); nindimm@gmail.com (M.M.N.)

**Keywords:** amino, acids, sericin, crosslink, X-ray diffraction, moisture vapor transmission rate, swelling, water vapor permeability, degradation, antibacterial

## Abstract

The application of silk sericin as a polymeric biomaterial has recently gained interest, although its film was found to be fragile, exhibiting brittleness when subjected to relatively slight stress, and it also displayed higher water solubility. This study focused on the enhanced physico-mechanical properties of the three films obtained by the crosslinking of sericin protein from three silkworm cocoons with poly (vinyl alcohol) (PVA) to reduce phase separation and solubilization of the films by promoting miscibility between sericin and PVA. The findings demonstrated how crosslinking with glutaraldehyde enhanced thermal stability and tensile strength and controlled the solubility of the three sericin–PVA films. The sericin from *G. postica*, *G. rufobrunnea*, and *Argema mimosae* is composed of serine, aspartic acid, and glutamic acid, which make up 80% of the total polar amino acids. X-ray diffraction (XRD) patterns showed that sericin–PVA films have semicrystalline features, representing amorphous and crystalline regions. The XRD results also indicated that the *Saturniidae* sericin–PVA film (Sat-SPF), *Gonometa postica* sericin–PVA film (GP-SPF), and *Gonometa rufobrunnea* sericin–PVA film (GR-SPF) have crystallinity percentages of 66.4%, 55.9%, and 17.7%, respectively. The moisture vapor transmission rate (MVTR) values observed in this study ranged from 991.2 to 5160 g/m^2^/24 h, indicating that these films can effectively regulate moisture levels in wounds. The swelling capacity of the three sericin–PVA composite films depends on the crosslinking density of their structures and was also found to be sensitive to the pH of the aqueous media, demonstrating their hydrophilic nature and potential use in drug delivery systems. The water vapor permeability of sericin–PVA films increased with higher environmental relative humidity (RH) and moisture content within the films. The elongation at break for GP-SPF (107.2% ± 3.1) and Sat-SPF (73.0% ± 4.1) was significantly higher than in GR-SPF (29.3% ± 2.3). However, their tensile strength and elastic modulus were lower than those of GR-SPF. These results show that the number of polar groups (amino and hydroxyl groups) from both sericin and PVA influences all the properties of the sericin–PVA composite films. The three sericin–PVA solutions were found to have antibacterial efficacy against three Gram-positive and one Gram-negative bacteria over 24 h. Scanning electron microscopy (SEM) images revealed a rough surface with a granular network pattern, which supports the potential use of sericin–PVA films for cell adhesion and proliferation, which are essential for biomedical wound dressing applications.

## 1. Introduction

Natural polymeric-based biomaterials offer a diverse range of chemical, physical, and mechanical properties by modifying their chemical structure and functional characteristics through the blending of various functional groups with low- and high-molecular-weight chains [1]. Natural polymers, also known as biopolymers, can support cell adhesion, migration, proliferation, and differentiation of cells in the surrounding environment. These biopolymers are known to induce extracellular matrix formation and stimulate tissue repair, making them suitable for tissue engineering applications [2]. Biopolymers derived from polysaccharides and proteins have been utilized in various medical applications due to their high biocompatibility, biodegradability, non-toxicity, and potential for biological recognition [3]. This is because their chemical structures and compositions are analogous to the macromolecules of the native extracellular environment, which supports their wound dressing applications [4].

An example of such a biopolymer is inexpensive silk sericin, a globular protein produced during the degumming process in the silk textile industry. Sericin protein is considered a by-product that is discarded when degumming wastewater [5]. However, it has been found to possess important biochemical properties. Silk sericin consists of 18 amino acids that include amino, hydroxyl, and carbonyl groups in their side chains [6]. Silk sericin utilizes these functional groups to crosslink, blend, and copolymerize with other polymers to create improved biomaterials with enhanced properties. Its hydrophilic nature is attributed to the high content of polar amino acids, specifically serine, aspartic acid, and glutamic acid. These polar amino acids confer bioactive properties upon silk sericin, such as antioxidant, antibacterial, anti-tyrosinase, UV-B resistance, moisturizing ability, and solubility in hot water [7].

The versatility of sericin as an ideal biopolymer for biomedical applications is based on the finding that it has a mitogenic effect on mammalian cells, which further supports the potential use in cell culture and tissue engineering [8,9]. Skin repair application of sericin films was supported by slow degradation, which heightened fibroblast cell attachment, promoting cell viability [10]. It was found that crosslinking sericin and PVA influences chemical structure changes, accordingly affecting the swelling degree and contact angle of the sericin–PVA film [11]. The feature indicates that the sericin–PVA composite films can be utilized for wound healing and topical drug release.

Several studies have reported the development of silk sericin composites into suitable biomedical scaffolds, including nanofibers, films, hydrogels, membranes, nanoparticles, and porous 3D sponges [12,13,14,15,16,17,18]. Sericin materials and composites have been used in several tissue engineering applications, including skin care, as drug delivery vehicles, and as cell culture additives [19,20,21,22,23,24,25]. For the relevance of this study, a brief review is provided on the effectiveness of silk sericin composite films in supporting wound healing, tissue engineering, and drug delivery systems. For example, the efficacy of sericin extract as a wound healing component was highlighted in a study by Tsubouchi and colleagues (2005) [26]. The study found that sericin enhanced the attachment of primary cultured human skin fibroblast cells, and this promotion of attachment and subsequent proliferation of skin fibroblasts is considered crucial in the healing process of skin lesions [26]. In a similar study by Teramoto and colleagues (2008) [27], sericin solution was gelled with ethanol to form gel films. Infrared results showed that these sericin gel films consisted of water-stable β-sheet networks assembled during gelation. The findings demonstrated that the sericin gel film could rapidly absorb water and reach an equilibrium water content of 80%. Additionally, the results showed that mouse fibroblasts adhered to and exhibited no cytotoxicity when placed on the sericin gel film. These attributes highlight the potential of sericin film for wound dressing [27]. The sericin films produced by Nayak and colleagues (2012) showed slow degradation, increased fibroblast cell attachment, and high cell viability, making them effective for skin repair applications [10]. Other studies also demonstrated the healing ability of sericin film, as it stimulates the migration and proliferation of different cell lines, including mammalian and hybridoma, as a supplement in serum-free media [28,29]. Sericin can also promote healing by activating collagen synthesis, improving the adhesion of cultured human skin fibroblasts, and further enhancing corneal wound healing. It has been shown that sericin’s wound healing ability is linked to an amino acid with antioxidant activity. Methionine plays a crucial role in stimulating collagen synthesis during wound healing [8,30,31,32]. Additional characteristics of sericin, such as moisture retention factor, antimicrobial activity, and enhanced oxygen permeability, are also important parameters for normal and accelerated wound healing [33,34,35].

Although sericin possesses beneficial properties, its poor mechanical properties and highly hydrophilic character in an aqueous environment limit its application in various biomedical applications. The challenge stems from its amorphous structure and low molecular weight, which result from degradation during the harsh degumming process. Sericin protein alone produces fragile materials that are not suitable for biomedical applications. It cannot form a polymeric film by itself because it becomes brittle and difficult to peel off the cast platform when dry. However, its chemical composition, especially reactive functional groups (amino, carboxyl, and hydroxyl), gives it the potential to react with both natural and synthetic polymers [36,37]. Therefore, silk sericin is subjected to a variety of modifications by blending or crosslinking with many other biopolymers and synthetic polymers to produce films with improved mechanical and physical properties, making it suitable for biomedical applications [38]. Polyvinyl alcohol, a synthetic polymer that is hydrophilic due to its high number of hydroxyl groups, was used to form the structural backbone of the sericin films [39,40]. Therefore, silk sericin was incorporated into polyvinyl alcohol via crosslinking with glutaraldehyde to form sericin–PVA films with improved physical and mechanical properties.

The findings of this study will provide in-depth evidence of how factors such as polarity, crystallinity, crosslinking density, free volume, and intermolecular interactions affect the internal structure of sericin–PVA composite films, which often influence both physico-mechanical properties simultaneously. Although these properties are not directly interrelated, this study presents evidence of how the material composition of sericin–PVA composite films affects both physical strength and water vapor permeability. In addition, the study presents evidence of the suitability of sericin–PVA composite films as ideal wound dressings, with characteristics including cell attachment and antibacterial efficacy, which are required for accelerated wound healing and protection. Other advantages of sericin–PVA composite film wound dressings include the sericin protein, which mimics the structure of the ECM, pH response, swelling ability, and controlled absorption of exudate, which maintains wound moisture and promotes cell adhesion and growth.

Although several studies have been conducted on silk sericin, to the best of our knowledge, there is no information available about sericin–PVA as a composite film for possible wound dressing applications. Therefore, many reported biomedical studies of silk sericin as a biomaterial focus on the development of hydrogels and nanoparticles. Furthermore, this study will highlight the potential biomaterial properties of these native Southern African sericin extracts for wound dressing and drug delivery systems.

## 2. Results

### 2.1. Amino Acid Composition

The results of Table 1 show the composition of major amino acids that are present in high amounts in the three sericin extracts. The results show that in each of the sericin extracts, serine, aspartic acid, and glutamic acid constituted 80% of the total polar amino acids. These polar amino acids render the three sericin extracts largely hydrophilic and acidic. The isoelectric points of the three sericin extracts were around pI 4.0, which is a result of the higher number of acidic amino acids compared to basic amino acids. These distinctive attributes are mainly due to the high number of amino, carboxyl, and hydroxyl groups, which are accessible during functional modifications. For example, arginine and lysine are the two amino acids known to donate their amines for crosslinking during gelation [41]. Also, serine is known to play a major role in stabilizing the protein structure by forming hydrogen bonds with the neighboring backbone amino and carboxyl groups. Therefore, the high serine content in the three sericin proteins means that their chemical structure will facilitate crosslinking with PVA polymers.

Furthermore, the three sericin extracts in this study were found to have both glycine and alanine in high concentrations, though low compared to their fibroin proteins. Glycine can fit into hydrophilic and hydrophobic environments because of its minimal side chain, consisting of just one hydrogen atom [42]. It also acts like polar amino acids, whose functional groups are available and influence the structure and solubility of the sericin films.

### 2.2. X-Ray Diffraction of Sericin–PVA Films

**Figure 1:**
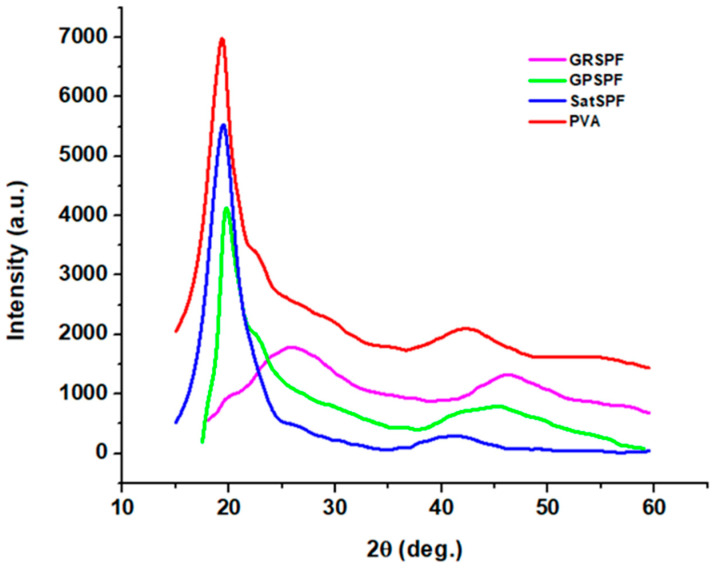
X-ray diffractograms of GP-SPF, GR-SPF, and Sat-SPF films.

The results shown in Figure 1 reveal the secondary structural transitions of three sericin–PVA films (GR-SPF, GP-SPF, and Sat-SPF), characterized using X-ray diffraction spectroscopy. The diffraction peaks of GP-SPF and Sat-SPF films at approximately 2θ = 19.9° and 2θ = 20.1°, respectively, appear as a combination of two features: one containing random coils and β-sheets, indicative of amorphous and crystalline regions of sericin protein. The other feature shows a sharp, high-intensity PVA peak, reflecting crystallinity due to hydroxyl groups in its side chains [43]. Crosslinking of sericin and PVA with glutaraldehyde caused a transition from β-sheet structures to a random coil form, driven by intermolecular hydrogen bonding. Additionally, characteristic crystalline PVA shoulder peaks were observed for GP-SPF and Sat-SPF films around 2θ = 46.5° and 41.7°, respectively.

GP-SPF and Sat-SPF films contain a high level of polar amino acids with hydroxyl (–OH) and amino (–NH) groups in their side chains. During crosslinking with glutaraldehyde, not all polar groups from PVA and sericin react. Consequently, both GP-SPF and Sat-SPF exhibit a semi-crystalline structure characterized by distinct, high-intensity peaks related to partial crystalline diffraction and an amorphous region.

However, in the case of the GR-SPF film, a single broad flat peak of low intensity at around 2θ = 26.4° and a characteristic PVA shoulder peak at around 2θ = 46.4° were observed. These peaks are a result of a shift because of a merger between the PVA peak at 2θ = 19.6° and the peaks of sericin at around 2θ = 19.9° and 24.0°, which means the number of hydroxyl and amino groups for both compounds is either decreased or depleted, initiating a decrease in the crystallinity of the films. Consequently, the peak intensity of the GR-SPF film was much lower than the peak intensities obtained for the other two films.

Additionally, Table 2 shows that the crystallinity percentage of the GR-SPF film is lower than that of the GP-SPF and Sat-SPF films. As mentioned earlier, the decrease in crystallinity results from the crosslinking density within the GR-SPF film, which encourages a randomly disordered amorphous structure, leading to a broad peak. Sat-SPF exhibits a higher percentage of crystallinity compared to GP-SPF film. The Argema mimosa sericin used in the Sat-SPF film displays shoulder peaks around 2θ = 14.2°, 30.6°, and 32.1°, along with a major diffraction peak divided into three smaller peaks at 2θ = 20.0°, 20.8°, and 21.4°. These peaks indicate a large β-sheets structure from sericin, contributing to the high crystalline percentage in Sat-SPF film. Conversely, in *G. postica* sericin, shoulder peaks are absent, and only a main peak at 2θ = 20.4° is observed [40]. This suggests that its crystalline contribution mainly derives from a high number of hydroxyl (–OH) groups present in both PVA and sericin protein. So, not all polar groups reacted during crosslinking with glutaraldehyde. Therefore, the number of polar groups in PVA and sericin influences crosslinking, which in turn shapes the structure of the sericin–PVA films.

### 2.3. Moisture Vapor Transmission Rate and Thickness of Sericin–PVA Films

The moisture sorption properties of sericin–PVA films are crucial in demonstrating their suitability as biomedical materials. Table 3 shows the moisture vapor transmission rate when two saturated salt solutions, KCl and MgCl_2_, were used for humidity control. The MVTR values obtained in this study range from 991.2 to 5160 g/m^2^/24 h for KCl and 1320 to 2916 g/m^2^/24 h for MgCl_2_. The MVTR values for the GR-SPF film demonstrate how increasing the GA concentration reduces the MVTR. This decrease may be attributed to the limited availability of polar groups in its sericin and PVA. During crosslinking, glutaraldehyde links the polar amino (–NH_2_) and hydroxyl (–OH) groups of sericin with the hydroxyl (–OH) groups of PVA. As a result, fewer polar groups are available for water vapor attachment, resulting in a lower MVTR. This suggests that the MVTR depends on the number of polar groups (amino and hydroxyl) from both sericin and PVA. These findings align with the number of polar amino acids found in the three sericin extracts (in Table 1). Hence, Sat-SPF films and GR-SPF films had lower MVTRs compared to GP-SPF films, which are constituted by a high number of polar groups. The MVTR results confirm that the crosslinking and the number of polar groups in a sericin–PVA composite film determine the interaction of water vapor and the chemical structure of the film.

Moreover, glycerol was added as a plasticizer during film formation to enhance flexibility and prevent the films from becoming brittle and fragile. It should be noted that the presence of the hydroxyl groups belonging to glycerol contributes to an increase in the films’ hygroscopic nature, which further influences the MVTR of the composite films [44]. To ensure uniformity in this study, 1% glycerol was added to all films, ensuring that the physical difference it makes remains constant across all experimental groups. This allowed the study to focus on how other factors affect the resulting film properties.

The thickness of sericin–PVA composite films shown in Table 3 varies depending on the percentage of GA used for crosslinking sericin and PVA. Films crosslinked with 3% glutaraldehyde ranged from 0.052 to 0.066 mm. The results in Table 2 also show that the thickness of GP-SPF, GR-SPF, and Sat-SPF films increases as the amount of crosslinking agent increases, while their moisture content decreases. Both GP-SPF and GR-SPF films were thinner and more flexible than the Sat-SPF films. Although Sat-SPF films were thicker and slightly flexible, this may be due to external environmental factors affecting the samples. In particular, the presence of pigments (dark brownish) within the solutions is believed to play a significant role during the annealing process of molded sericin–PVA films [45]. For the two Gonometa sericin films, it was found that the small amount of pigment (pale yellowish) had little effect on their film thickness.

### 2.4. Swelling Degree of Sericin–PVA Films

**Figure 2:**
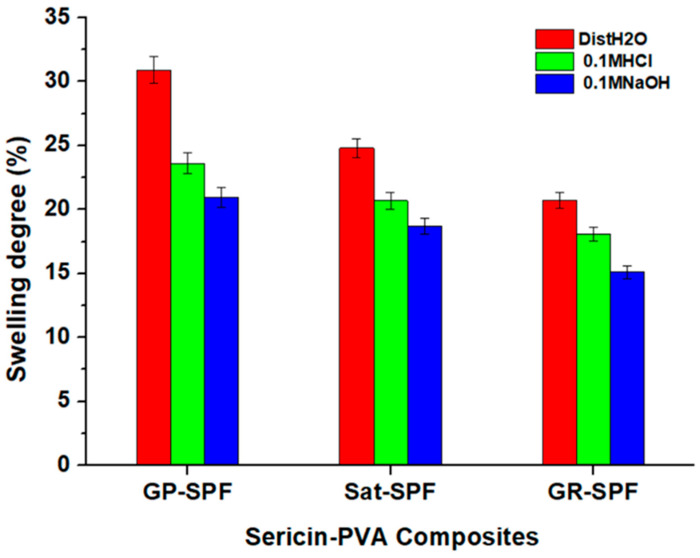
Swelling degree of GP-SPF, GR-SPF, and SAT-SPF films after immersing in Dist. water, 0.1 M NaOH, and 0.1 M HCl.

**Figure 3:**
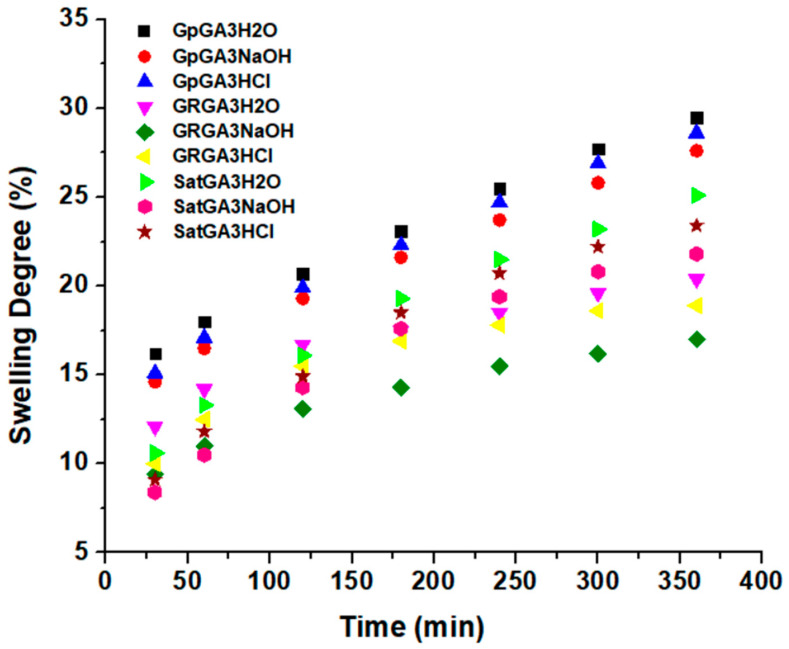
Swelling degree percentages of GR-SPF, GP-SPF, and Sat-SPF, when immersed in the media over time.

The swelling degree of a composite film is an important property useful for defining the chemical structure and the crosslinking density of polymeric networks. The results of Figure 2 show the swelling response of the three sericin–PVA films crosslinked with 3% GA. The results of the three sericin–PVA films after immersing them in distilled water (neutral), 0.1 M NaOH (alkaline), and 0.1 M HCl (acidic) for 6 h are illustrated in Figure 3. It was found that during the first 3 h immersion, the volume of the sericin–PVA films increased to the equilibrium of the final maximum swollen volume faster in all three tested media pH conditions. This fast phase was followed by a steady, slow swelling degree of the films, indicating that compact crosslinked networks characterize their structures. Such a structure with a high crosslinking density results in smaller spaces within the molecules available for the water interaction. The results of the GP-SPF film showed a higher degree of swelling when immersed in the three media pH conditions for 6 h. The highest swelling degree was observed in distilled water, followed by 0.1 M NaOH and 0.1 M HCl, respectively. This is due to osmolarity and the available space resulting from the high number of polar groups still capable of interacting with the media after crosslinking. Osmolarity encourages swelling in GP-SPF films by creating an osmotic pressure difference between their interior (which contains polar groups) and the surrounding pure water. As a result, it exhibited a higher swelling ratio than both GR-SPF and Sat-SPF films across all media. In 0.1 M NaOH and 0.1 M HCl solutions, the external osmolarity is higher, reducing the osmotic gradient and leading to a lower overall swelling degree compared to distilled water. Therefore, the magnitude and direction of swelling are directly proportional to the difference in osmotic pressure between the two sides of the film.

Furthermore, from the result of the GP-SPF film, the media absorption into the film network continued beyond the 3 h immersion period, in contrast to what was observed as a swelling equilibrium point for both GR-SPF and Sat-SPF films, which is considered fast.

Then, the swelling rate became slower, indicating a steady state, which is attributed to the gradual depletion of the polar groups that occurred during crosslinking, leading to a restriction of space within the film networks. In addition, it also confirms that the acidic or basic groups within sericin–PVA films either accept or donate protons, depending on the environmental pH to which they are subjected. Therefore, sericin–PVA composite films have great potential to be used as medicated wound dressing materials in the wound healing process.

### 2.5. Water Vapor Permeability of Sericin–PVA Films

**Figure 4:**
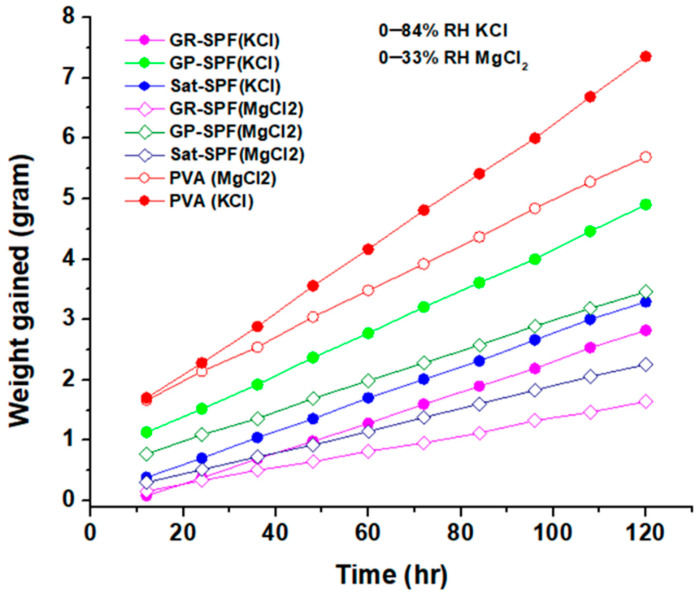
Illustration of mass variation of three sericin–PVA films that were incubated in two salt solutions (KCl and MgCl_2_) to control the relative humidity of a desiccator over time.

The results of Figure 4 show the mass variation of a desiccant (CaCl_2_) when the flux of water vapor passes through three sericin–PVA composite films (GP-SPF, GR-SPF, and Sat-SPF) used in sealing the cup. The data obtained on mass gain relates to a set of measurements performed over five days using the gravimetric cup method. Figure 4 presents the mass gain curves when sericin–PVA films were incubated at 30 °C in saturated solutions of MgCl_2_ and KCl, conditioned at relative humidities of 0–33% and 0–84.4% RH, respectively. The observed linear mass increase reflects the recorded water vapor flux through the serici-nPVA films at 12, 24, 36, 48, 60, 72, 84, 96, 108, and 120 h. Pure PVA film was used as a control to compare its water vapor flux, and that of the sericin–PVA composite films. The obtained mass gain of PVA film in both saturated solutions exhibits a highly hydrophilic structure with many hydroxyl (–OH) groups on its chain that attract water molecules, allowing them to pass through easily, which may lead to swelling. The linearity of the curves is evidence of a pre-conditioning of the sericin–PVA films before the start of the experiments. This process was undertaken to mitigate hysteresis effects by stabilizing the polymeric material’s properties and lessening undesirable interactions, resulting in a more uniform response to the cycled stimuli [46].

**Figure 5:**
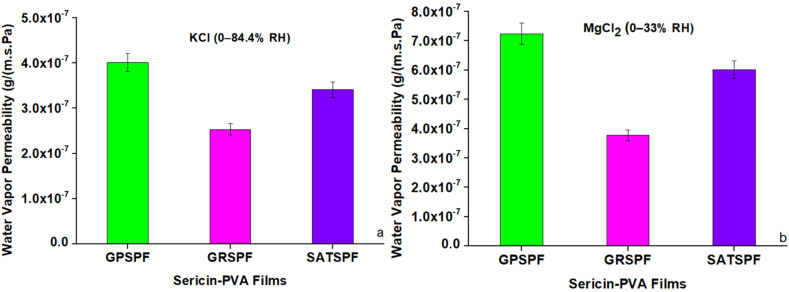
Water vapor permeability graphs of three sericin–PVA composite films that were incubated in two saturated solutions ((**a**) KCl and (**b**) MgCl_2_) to control the relative humidity of a desiccator over time.

Figure 5a,b presents the results of water vapor permeability of the three sericin–PVA composite films (GP-SPF, GR-SPF, and Sat-SPF) when exposed to saturated solutions of KCl and MgCl_2_. The obtained water vapor permeability results show similar behavior among the three sericin–PVA films, though they were exposed in two different systems. Figure 5b presents the result of water vapor permeability when a saturated MgCl_2_ solution was used. From its results, the GP-SPF film has the highest water vapor permeability, followed by the Sat-SPF film, and the GR-SPF film produces the least. In Figure 5a, a similar sequence of water vapor permeability was also obtained when a saturated KCl solution was used. The high-water vapor permeability observed in both systems for the GP-SPF and Sat-SPF films is attributed to the film’s hydrophilic nature. This is due to the high number of polar groups in its sericin protein and the –OH groups of PVA, which can interact with water vapor.

The results from the saturated MgCl_2_ solution reveal higher water vapor permeability in the three sericin–PVA films compared to when exposed to the saturated KCl solution. This is due to the saturated MgCl_2_ solution’s enhanced ability to reduce water vapor pressure, generating a more potent driving force for water vapor transport through the sericin–PVA films from the solution side to the air side. This outcome is attributed to a number of factors, such as MgCl_2_’s higher solubility, its dissociation into more MgCl_2_ ions (Mg^2+^ and 2Cl^−^) in solution than KCl, which produces only two ions (K^+^ and Cl^−^), the larger osmotic pressure it generates, MgCl_2_’s ability to form more complex hydrates, and MgCl_2_ interacting with the sericin–PVA matrix and altering the structure of the films [47,48].

### 2.6. In Vitro Degradation of Sericin–PVA Films

**Figure 6:**
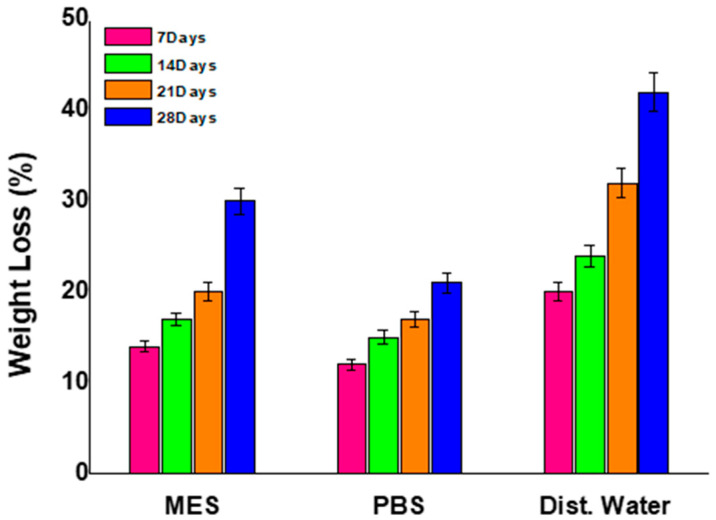
Weight loss (%) pattern of *G. postica* (GP-SPF) film when degraded in PBS, MES, and distilled water.

**Figure 7:**
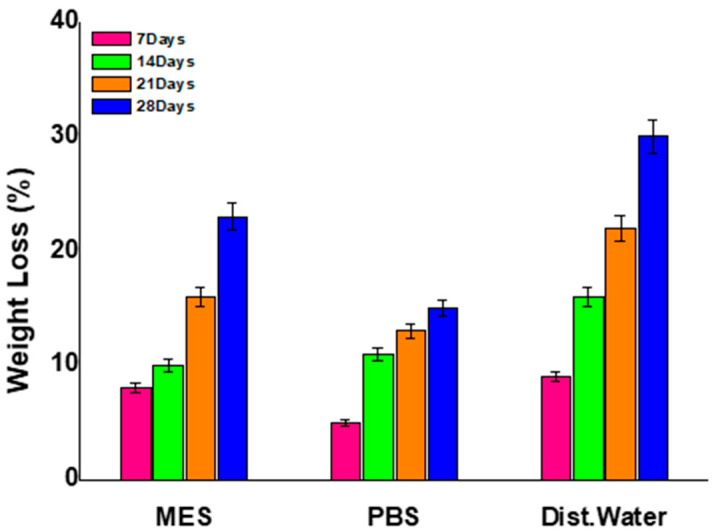
Weight loss (%) pattern of *G. rufobrunnea* (GR-SPF) film when degraded in PBS, MES, and distilled water.

**Figure 8:**
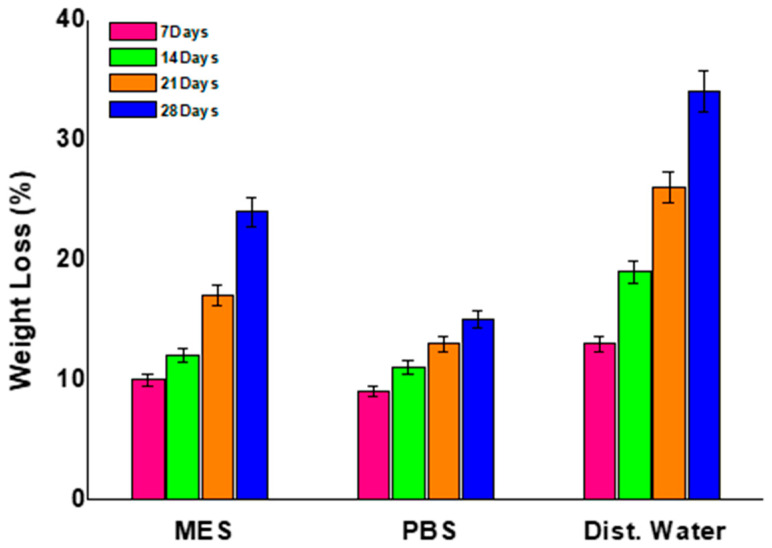
Weight loss (%) pattern of *Argema mimosae* (Sat-SPF) film when degraded in PBS, MES, and distilled water.

Figure 6, Figure 7 and Figure 8 present the results of the weight loss of the three sericin–PVA composite films (GP-SPF, GR-SPF, and Sat-SPF) after degradation in distilled water, PBS, and MES, along with 0.1% sodium azide solution. The obtained results demonstrate how degradation by distilled water presented the fastest and highest weight loss in all three sericin–PVA composite films. The degradation rates of the three sericin–PVA films in PBS were found to be slow and had lower weight loss compared to distilled water and MES. However, the results of the MES degradation rate indicate an intermediate weight loss in all three sericin–PVA films. The results of the GP-SPF film (Figure 6) show that after 28 days, 44% weight loss was observed for distilled water and 30% and 21% for MES and PBS, respectively. After 28 days, the degradation process of the Sat-SPF film (Figure 8) resulted in 35% weight loss when immersed in distilled water, and 25% and 17% weight loss for MES and PBS, respectively. In the case of GR-SPF film (Figure 7), the 28 days of degradation resulted in 30% weight loss when immersed in distilled water, and 23% and 15% for MES and PBS, respectively. It was found that after 7–28 days, the degradation rates of the three sericin–PVA films in all the media showed the highest weight loss for the GP-SPF film, followed by the Sat-SPF film, with the lowest weight loss observed in the GR-SPF film. From the findings of Figure 6, Figure 7 and Figure 8, the degradation of all three sericin–PVA films is suited for drug delivery and tissue engineering applications, as polymeric composite materials may be required to degrade within a timeframe that is comparable to tissue healing, and they can regulate the drug release rate at a specific site.

### 2.7. Mechanical Properties of Sericin–PVA Films

The results of Table 4 present the mechanical properties of three sericin–PVA composite films (GP-SPF, GR-SPF, and Sat-SPF). Mechanical properties, such as tensile strength (TS), elastic modulus (EM), and elongation at break (% E), are highlighted to demonstrate the films’ durability for potential biomedical uses. It is noted that films made from pure sericin lack excellent mechanical properties due to their fragility and tendency to swell or dissolve easily in water at high temperatures [49]. Therefore, to improve sericin films, crosslinking of pure sericin with PVA was used to enhance the mechanical properties of the sericin–PVA composites. Crosslinking sericin and PVA with glutaraldehyde (GA) adds structural strength to the films, as the tensile test results in Table 4 indicate that increasing the GA concentration raises the tensile strength and elastic modulus of the sericin–PVA films. However, it was found that the elongation at break decreases as GA content increases. The rise in tensile strength and elastic modulus results from the interaction between GA, PVA, and sericin chains during crosslinking, which creates more ether linkages [38]. Crosslinking offers some key benefits to the sericin–PVA films, but using high concentrations of GA makes the films stiffer due to restricted polymer chain mobility [50]. To address the stiffness, 1% glycerol was added as a plasticizer to improve flexibility, though this significantly decreased the tensile strength and elastic modulus of the films. Adding glycerol also increased the elongation at break for all three sericin–PVA films, especially in the cases of GP-SPF and Sat-SPF films. The elongation at break for both GP-SPF (107.2% ± 3.11) and Sat-SPF (73.0% ± 4.1) films was high compared to the GR-SPF film (29.3% ± 2.32). However, their tensile strength and elastic modulus were found to be low compared to the GR-SPF film.

### 2.8. SEM of Sericin–PVA Films

**Figure 9:**
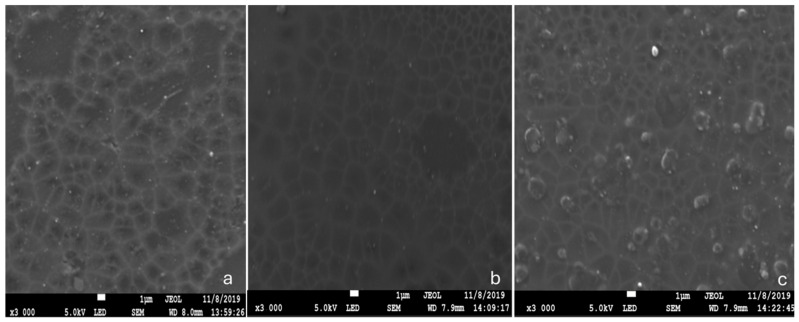
Scanning electron microscopy images of the three sericin–PVA films: (**a**) *GP-SPF*, (**b**) Sat-SPF, and (**c**) GR-SPF.

The morphology test of the three sericin–PVA films was analyzed by scanning electron microscopy (SEM). The SEM image (magnified 3000×) in Figure 9 provides information about the surface morphology belonging to (a) *GP-SPF*, (b) Sat-SPF, and (c) GR-SPF films. The three films were found to have a particular network pattern, with the number of granules embedded within the film surfaces. In the GR-SPF film, this network was uniformly distributed throughout the film surface, suggesting that crosslinking with glutaraldehyde promoted the miscibility of PVA and sericin, resulting in decreased phase separation compared to GP-SPF and Sat-SPF films, which had some of the network segments showing a small part of a surface that is different from the area around. The phase separation observed in GP-SPF and Sat-SPF films is due to a shortage of GA, which was completely consumed during the linkage between sericin and PVA. As a result, some of the polar groups from sericin and PVA were left behind without being crosslinked. The results show that the amount of GA plays a vital role in crosslinking polar groups such as amino, carboxyl, and hydroxyl, which normally occur outside the random coil in a hydrophilic environment. Similar findings of phase separation were also observed in other studies [51]. In addition, all three films presented rough surfaces that consist of small-sized embedded spherical particles of a nanoscale, creating more surface area and better adherence for cell culture attachment.

### 2.9. Sericin–PVA Antibacterial Capacity

To evaluate the antibacterial activity of the sericin–PVA solutions, this study focused on their interaction with the cell walls of the Gram-positive and Gram-negative bacteria. This was achieved by optimizing the pH of sericin–PVA solutions to determine their effect on the antibacterial properties of the three sericin–PVA solutions. The results of Table 5 show the antibacterial activities of three sericin–PVA solutions at a concentration of 10 mg/mL and a pH of 3. The findings are consistent with previous studies, which indicate that Gram-positive bacteria are more susceptible to sericin–PVA solutions, while Gram-negative bacteria (Escherichia coli) showed a weak inhibition zone around the agar wells. The GP-SPF solution presented higher inhibition of bacterial growth, with zones measuring approximately 18.45, 18.90, and 19.63 mm for B. subtilis, S. aureus, and S. epidermidis, respectively. Both GR-SPF and Sat-SPF solutions showed a similar pattern of inhibition against the three Gram-positive and one Gram-negative bacteria. However, their effectiveness appears to differ in their ability to produce a bacteriostatic effect. The variation in antibacterial activity among the three sericin–PVA solutions likely depends on the total number of amino acids with positively charged side-chain groups present in each film [7]. These findings demonstrate the antibacterial effectiveness of the three sericin–PVA film solutions against three Gram-positive and a weak inhibition on Gram-negative bacteria over 24 h.

## 3. Discussion

The results of this study show how the three fabricated sericin–PVA composite films have features similar to other composite films made from common biopolymers (chitosan, fibroin, collagen, gelatine, alginates, etc.) [52,53]. Similarities were seen during the characterization of the chemical, physical, mechanical, and biological properties of the three sericin–PVA films. Regarding chemical structure, the sericin protein in each of the three composite films was found to have a high content of polar amino acids (see Table 1), which are important for crosslinking and modifying their functional properties. The number of polar amino acids in the three sericin proteins was similar to what has been reported in the literature, where sericin protein mainly consists of polar amino acid groups, such as serine, aspartic acid, and glutamic acid, which contain aliphatic hydroxyl groups [7]. According to previous studies, this high number of polar amino acids explains why sericin exhibits water absorbability and good solubility [54]. Looking at the secondary transition structure, the findings from this study’s X-ray diffraction spectroscopy align with other research showing diffraction peaks that have semicrystalline features, indicating both amorphous and crystalline regions. The two main factors affecting the chemical structure of sericin–PVA films are the number of polar amino acids and the crosslinking density between sericin and PVA. For instance, the crystallinity percentage results (see Table 2) confirm that a high crosslinking density in sericin–PVA films increases the amorphous region while decreasing the crystalline structure in films with fewer polar groups [50]. The broad diffraction peak observed in a highly crosslinked GR-SPF film suggests it is more amorphous due to the reduction or depletion of hydroxyl networks that are responsible for crosslinking, crystallinity, and water interaction. Similarly, Chen and colleagues explained that the reduction or loss of crystallinity is caused by crosslinking in chitosan–gelatine films because of decreased hydrogen bonding in chitosan molecules, resulting in an amorphous structure for the polyelectrolyte complex [53]. This highlights the importance of polar groups (like –OH groups in PVA and sericin) that form hydrogen bonds, promoting crystallization. If a film has fewer of these polar groups, the ability for such ordering decreases, further reducing crystallinity when high crosslinking occurs [55]. The high-intensity pure PVA film diffractogram represents a mixture of crystalline and amorphous regions, with a characteristic peak at 2θ ≈ 19.4° as its most prominent feature. These biochemical features endow silk sericin with important biological properties, such as biodegradability, biocompatibility, and moisture retention, among others [56,57].

The results from the chemical structure analysis of the three sericin–PVA films demonstrate how the number of polar groups and crosslinking affect their physical properties, including the moisture vapor transmission rate (MVTR), swelling degree, and water vapor permeability (WVP). The MVTR results in Table 3 demonstrate the moisture absorption capacity of these sericin–PVA composite films. Under both saturated salt solution humidity conditions, GP-SPF films show the highest MVTR due to the highest number of polar groups, followed by Sat-SPF films, with GR-SPF films having the lowest MVTR. The MVTR values in this study suggest that the three sericin–PVA films are comparable to the reported WVTR values of intact skin, which range from 240 to 1920 g/m^2^/24 h, while an uncovered wound has a WVTR of approximately 4800 g/m^2^/24 h, and others have observed a WVTR of approximately 10 times more than that of intact skin for freshly excised wounds [58,59]. Another study by Wu and colleagues found that water vapor loss mainly depends on wound depth, with MVTRs of 427, 1480, and 1953 g/m^2^/24 h for superficial, deep partial-thickness, and full-thickness burns, respectively [60]. The WVTR of the three sericin–PVA composite films observed in this study ranged from 991.2 to 5162 g/m^2^/24 h, similar to that of intact skin and burns of different depths. Furthermore, the MVTR of GP-SPF is comparable to that of an uncovered wound, which can prevent wound desiccation and maintain a sufficiently moist environment for healing. These results not only indicate the effective moisture regulation of these films but also their potential for wound healing and drug delivery applications, offering a promising future for biomaterials in these fields.

Similar to MVTR results, the swelling degree of GP-SPF films is the highest due to the highest number of polar groups, followed by Sat-SPF films, with GR-SPF films having the lowest swelling degree in all media. From the obtained results, the relationship between swelling degree and the MVTR of the sericin–PVA composite films reveals how its chemical structure modification plays a vital role in regulating the absorption of water vapor and swelling degree. The modification of the chemical structure through the crosslinking process and the number of available polar groups in sericin–PVA composite films influence the structure of swelling and moisture transport pathways within the film, providing crucial insights into the technical aspects of our research [61].

From the results in Figure 2, it is clear that crosslinking causes changes in the chemical structure, leading to stable, insoluble sericin–PVA composite films that swell when placed in distilled water (neutral), 0.1 M NaOH (alkaline), and 0.1 M HCl (acidic). The swelling behavior of sericin–PVA films provides strong evidence of a successful crosslinking process. The degree of swelling followed the order GP-SPF > Sat-SPF > GR-SPF, which relates to the crosslinking density and the number of polar amino acids present. The swelling percentages of GP-SPF, Sat-SPF, and GR-SPF films were 32%, 25%, and 20%, respectively, after 6 h of immersion in water. These findings suggest that sericin–PVA composite films have the potential to manage low exudates, due to their internal composite structure, which allows them to operate as effective dressings that quickly absorb excess exudates from wounds and reduce maceration of surrounding skin while maintaining high moisture levels at the wound site [62]. For instance, the 3 h immersion period marks the swelling equilibrium point for GR-SPF and Sat-SPF films, which is considered rapid for media absorption. In contrast, the media absorption of the GP-SPF film continued beyond the 3 h mark, meaning it has the potential to deal with deep partial-thickness wound burns. According to the existing literature, there is no definitive evidence that one dressing outperforms another in managing exudate or promoting healing.

Additionally, it is difficult to evaluate clinical differences in exudate management between various products due to the lack of a standard assessment method [63]. Therefore, the effectiveness of the dressing can be measured by its ability to rapidly absorb exudates and retain wound moisture. Additionally, it is clear from Figure 2 that the pH of the surrounding media also affects the swelling behavior of the sericin–PVA composite films. For instance, when immersed in three aqueous media—distilled water, 0.1 M HCl, and 0.1 M NaOH—for a specific period, the swelling degrees were highest in distilled water, followed by 0.1 M HCl, with the lowest swelling in 0.1 M NaOH [64,65]. Since sericin protein contains amino and carboxyl groups, along with other acidic and basic groups in its side chains, the high swelling in distilled water occurs because water’s amphiprotic nature allows it to act as an acid or a base, protonating or deprotonating the amino acids’ carboxyl (-COO^−^) and amino (–NH3^+^) side chains on the films. This means the hydrogen ions from water protonate the amino groups on the sericin side chains. These positively charged ammonium groups (–NH3^+^) create electrostatic repulsion between molecules, causing them to repel each other and increasing the film’s water absorption. Similarly, in 0.1 M HCl, the films gain a net positive charge due to the protonation of all basic amino groups (–NH3^+^) on the sericin–PVA side chains, which enhances hydrophilicity and swelling. He and colleagues reported similar results [66]. In the case of 0.1 M NaOH, the films develop a net negative charge because the deprotonation of acidic side chains on amino acids, such as the carboxyl groups of aspartic acid and glutamic acid, leads to the loss of protons (H^+^), resulting in negatively charged (-COO^−^) groups, as observed by Sung and colleagues [67]. Additionally, the swelling levels in 0.1 M HCl and 0.1 M NaOH aqueous media are related to how close the amino acid side chains are to their isoelectric points. Since the net charge of an amino acid side chain influences its electrostatic repulsion, when the pH is far from the pI, the side chains carry a net charge, leading to increased repulsion and swelling. The observed pH-responsive swelling behavior within the acidic-neutral range indicates that sericin–PVA films are suitable for drug delivery systems. Their ability to serve as a self-regulating carrier for bioactive agents in acidic conditions makes them especially useful for targeted drug delivery [68].

The water vapor permeability (WVP) of the three composite films was tested to predict their performance in environments with varying humidity and temperature. This helps understand their ability to maintain function under specified conditions. The microstructure of the sericin–PVA composite films, including factors like density, crystallinity, micro-fractures, and plasticizers, influences the WVP by affecting chemical interactions and physical structure, which in turn alter permeability. As shown in Figure 5a,b, the WVP results of the three films follow a pattern similar to the MVTR and swelling degree. For instance, the GP-SPF film has high WVP due to its abundant polar groups, followed by the Sat-SPF films, while the GR-SPF film exhibits the lowest permeability across all media. Therefore, the WVP ranking also depends on the degree of crosslinking within the sericin–PVA films and the differences in polar amino acid composition (see Table 1) of the sericin extracts. This shows that the microstructure and hydrophilic properties of sericin–PVA films influence how water solubility and diffusion occur through them, impacting their WVP [69,70]. Glycerol, a plasticizer added to improve film flexibility, also contributes to the hydrophilic nature of sericin–PVA films [71,72]. The effect of thickness on WVP aligns with existing research on hydrophilic films [73]. Films like SAT- SPF, which are thicker and have a larger surface area, display higher WVP compared to thinner GR-SPF films. This is because increased thickness creates a longer pathway for water molecules, promoting more contact points between water and the film’s hydrophilic groups, thereby enhancing solubility and diffusion. For the GP-SPF film, its elevated WVP is mainly due to a higher number of polar groups rather than its thickness. From the results, it is clear that water vapor permeability is not determined solely by thickness; factors such as temperature, humidity, and the material’s specific properties also play crucial roles [74,75,76].

From the degradation results (Figure 6, Figure 7 and Figure 8), the weight loss of all sericin–PVA films was below 50%, confirming the stability of the sericin–PVA composite films and illustrating how crosslinking sericin with PVA adjusts their degradation rate. This behavior illustrates how the chemical structure and degree of crosslinking in each of the three sericin–PVA films affect degradation rates [51]. For example, the degradation rates of the three sericin–PVA films followed a similar pattern to their swelling degrees (Figure 2), with the GP-SPF film exhibiting the highest swelling, followed by the Sat-SPF film, and then the GR-SPF film with the least swelling. These differences in swelling are attributed to variations in polar amino acid composition (Table 1) and the crosslinking density of each film. In the case of the GR-SPF film, the lower swelling indicates a compact, highly crosslinked structure. Such a structure with a high crosslinking density creates smaller spaces within the molecules, reducing water absorption and thus decreasing degradation of the film. Conversely, GP-SPF’s higher swelling capacity promotes greater hydrolytic degradation, resulting in higher weight loss. The Sat-SPF film shows intermediate weight loss, influenced by its crosslinking density and polar amino acid content.

These findings support the use of stable silk sericin as a promising component in composite biomedical materials, which could also be used for drug delivery due to its chemical reactivity and responsiveness to pH conditions [77]. Moreover, the degradation process of sericin–PVA films promotes cell adhesion and tissue regeneration by mimicking the natural extracellular matrix (ECM), providing a structural and functional environment for cells to attach, migrate, and proliferate. The degradation of sericin–PVA films releases signaling molecules (sericin protein) that stimulate cell growth and migration. This is especially important for cells like fibroblasts and keratinocytes, which are crucial for wound healing by increasing collagen synthesis and tissue regeneration [10,78].

The results of the mechanical properties (shown in Table 4) of the three sericin–PVA films depend on how GA forms hydrogen bonds between the –OH groups of glycerol, PVA, and the polar side chains (–NH_2_ and –OH) of pure sericin. This indicates that GA determines the size of the network structure in each film based on how completely it was consumed during the crosslinking reaction. Glutaraldehyde acts as a limiting reagent in this process, meaning that once it is fully utilized, the reaction stops, even if some polar groups from glycerol, PVA, or sericin remain unlinked to the network. This is demonstrated by the GP-SPF film, which contains higher numbers of polar amino acids with –NH_2_ and –OH groups in their side chains compared to Sat-SPF and GR-SPF films. For example, GP-SPF exhibits high elongation at break but lower tensile strength and elastic modulus. This supports the idea that some polar groups may not have formed crosslinks, leaving them available to interact with moisture or water. A decrease in tensile strength (TS) results from increased amorphous regions in the sericin–PVA films. Conversely, increased elongation at break suggests decreased elastic modulus due to enhanced flexibility in the films. Furthermore, GA crosslinking involves sericin’s amino groups from glycine and its simple side chain to create a dense, stable covalent network. Glycine’s small side chains produce minimal steric hindrance, allowing the sericin–PVA backbone to rotate freely and bend sharply at Gly residues, promoting a flexible yet compact internal structure. Along with extensive hydrogen bonding with PVA, this results in a robust, stable film with superior mechanical strength and durability [38,79]. These findings align with previous reports, emphasizing the role of GA crosslinking and the number of polar amino acid groups, mainly due to the high hydrophilicity of sericin molecules.

The surface morphologies of sericin–PVA films (Figure 9) were revealed by scanning electron microscopy, showing a rough surface with a small-particle-size network embedded within the film surfaces. The results indicate that the amount of GA plays a crucial role in crosslinking polar groups such as amino, carboxyl, and hydroxyl, which typically occur outside the random coil in a hydrophilic environment. This enhances the miscibility of PVA and sericin, reducing phase separation in composite films. Similar phase separation findings were also observed in other studies [51]. Additionally, all three films exhibited rough surfaces composed of small, spherical particles at the nanoscale, which increased the surface area and promoted better cell adherence. The results from this study also demonstrate the bacteriostatic properties of sericin–PVA solutions against four test bacteria (*Staphylococcus aureus*, *Staphylococcus epidermidis*, *Bacillus subtilis*, and *E. coli*). The antibacterial activity of the three sericin–PVA solutions aligns with their other investigated properties. For instance, the GP-SPF solution shows the highest antibacterial activity due to the greatest number of polar groups, followed by Sat-SPF and GR-SPF solutions. The superior antibacterial activity of GP-SPF compared to the other two is attributed to its high content of polar and non-polar amino acids (shown in Table 1), which confer high net charges and hydrophobicity. The three sericin–PVA solutions exhibit antimicrobial efficacy when the pH is below 4 because, at very low pH, the amino groups on sericin amino acids acquire a net positive charge (–NH3^+^) through protonation by excess hydrogen ions (H^+^), while the carboxyl groups (-COO^−^) also become protonated (-COOH). This enables interaction with the negatively charged bacterial cell wall via cationic groups, with hydrophobic groups mainly inserting into the cytoplasmic membrane, leading to leakage of proteinaceous and other intracellular constituents [80]. Although sericin’s antimicrobial action cannot be classified as that of antimicrobial peptides (AMPs), similar to cationic AMPs, it requires the modification of sericin amino acid moieties for functionality. Modified sericin exhibits notable physicochemical properties, such as low molecular weight, charge under low acidic conditions, hydrophobicity (with nearly 39% non-polar amino acids), and solubility [81]. The findings for sericin solution indicate a mechanism similar to those reported in the literature [7,33].

The findings demonstrate the essential role that these films can play in multifunctional drug delivery systems and wound dressings. This is confirmed by the overall findings of this study, which show that the three fabricated sericin–PVA films offer potential benefits for use in various biomedical applications. The following aspects will be investigated further: the use of sericin–PVA as a wound dressing capable of controlled drug release; the use of sericin protein for UV-A and UV-B resistance; and its natural moisturizing factors for skin application (NMF).

## 4. Materials and Methods

### 4.1. Materials

All the chemicals and reagents used were of analytical grade (95–99% purity): magnesium chloride, calcium chloride, potassium chloride, sodium chloride, sodium azide, glutaraldehyde (GA), glycerol, poly (vinyl alcohol) (PVA), 2-(N-morpholino)ethanesulfonic acid (MES) (≥99.5% (T)), phosphate-buffered saline (PBS), L-ascorbic acid (99% purity), and dimethyl sulfoxide (≥99% purity). Whatman syringe filters with a PVDF membrane (pore size 0.45 μm) were purchased from Sigma-Aldrich (Steinheim, Germany). The ultra-high purity (UHP) water used for all preparations was generated from a Milli-Q system with a resistivity of 18.2 MΩ cm (Millipore, Billerica, MA, USA). The three silk sericin samples were derived from three wild Southern African silkworm cocoons: The *Gonometa postica* cocoons were harvested in Northwest Province, South Africa, the Gonometa rufobrunnae cocoons were obtained in the Central District of Botswana, while the Argema mimosa cocoons came from the Manzini region in eSwatini.

### 4.2. Preparation of Sericin–PVA Films

To prepare the sericin–PVA films, approximately 0.4 g of polyvinyl alcohol (PVA) was weighed into vials and dissolved in a 7.0 mL solution of 3% (*v*/*v*) EtOH containing 1% (*w*/*v*) glycerol (plasticizer), which had been heated for 3 min in a microwave prior to use. To ensure complete solvation of PVA, the mixtures were stirred for one hour at 80 °C. The solutions were acidified by adding 1.0 mL of 0.05 N HCl and 1.0 mL of 3% (*v*/*v*) glutaraldehyde for crosslinking. The vials were stirred for 5 min before adding 1.0 mL of 3% silk sericin solution. The solutions were allowed to mix for 2 h at 90 °C to promote homogeneity. The crosslinked solutions were cast into glass Petri dishes and allowed to cool overnight at room temperature before being placed in an oven at 65 °C for 24 h to dry. The dried films were wetted with 70% ethanol to facilitate peeling off. The films were neutralized with distilled water and dried at 60 °C for 12 h before being stored in a desiccator for characterization.

### 4.3. Characterization of Films

#### 4.3.1. Amino Acid Analysis

About 18 amino acids that constitute silk sericin protein were obtained by hydrolyzing 10 mg of silk sericin samples in 6 M HCl at 110 °C for 24 h. This process was followed by derivatizing the 50 µL hydrolysates with Dabsyl-Cl in microcentrifuge tubes. The derivatized amino acid hydrolysates were dried and re-dissolved in ethanol. Separation and quantification were performed with an Agilent 1200 HPLC-DAD system (Agilent Tech., Waldbronn, Germany) equipped with Agilent ChemStation data software (version 4.3). The separation was achieved by running the derivatized amino acids through an Agilent Zorbax Eclipse XDR C18 (4.5 × 150 mm, 5 µM) column (Agilent, Santa Clara, CA, USA).

#### 4.3.2. X-Ray Diffraction Analysis of Sericin–PVA Films

The sericin–PVA films’ crystallinity was examined with a Rigaku Smart Lab 9 kW, high-resolution X-ray diffraction system (Rigaku, Neu-Isenburg, Germany) using CuKα radiation, to determine the diffraction intensity curves at a λ = 1.5 Å for 2θ from 10° to 60° at a scanning rate of 0.0015° s^−1^. The voltage and current of the X-ray source used were 200 mA and 45 kV, respectively. Furthermore, the percentage crystallinity of GP-SPF, GR-SPF, and Sat-SPF films was calculated from the relative integrated area of the crystalline and amorphous peaks through the following equations (Equation (1)) using Origin software (Origin 8.5.1):

(1) $$\%\;Cr=\frac{Acr}{\left(Acr+Aam\right)}\times 100$$

where Acr and Aam are the integrated areas of the crystalline and amorphous peaks after deconvolution of experimental patterns, respectively [82].

#### 4.3.3. Analysis of MVTR and Thickness of Sericin–PVA Films

The moisture vapor transmission rate (MVTR) capacities of sericin–PVA films were determined by placing them in different desiccators, where salt solutions controlled the relative humidity. Two saturated salt solutions of potassium chloride and magnesium chloride were used. The initial weight of dried films was obtained after drying in a vacuum oven for 2 h at 60 °C until a constant weight was achieved. The dried circular films (ID = 20 mm) were placed in desiccators with relative humidities of 84% (KCl) and 33.3% (MgCl) at 25 °C for 24 h. The films were then removed from the desiccators for weighing. All experimental measurements were performed in triplicate. The accurate thickness of the sericin–PVA films was measured using a digital micrometer (Vernier type; Mitutoyo, Tokyo, Japan) with an accuracy of 0.001 mm. The film thickness values are the average readings obtained after ten random measurements across each film specimen. The sericin–PVA film thickness measurements were recorded at 25 °C.

#### 4.3.4. Swelling Degree Analysis of Sericin–PVA Films

The swelling degree of the sericin–PVA films was determined following Mandal and coworkers’ method [38]. The films were conditioned for 12 h by placing them in an oven at 40 °C and then weighed using a Mettler Toledo balance ML series (Greifensee, Switzerland). Afterward, the dried films were immersed in 30 mL of distilled H_2_O, 0.1 N HCl, and 0.1 N NaOH for 4 h. After swelling, the sericin–PVA films were removed, and the excess solution was blotted with filter paper. The experiments were conducted in triplicate for each measurement. The percentage swelling of the sericin–PVA films at equilibrium was calculated using the equation below (Equation (2)),

(2) $$\%\;\mathrm{Swelling}=\frac{W_{sw}-W_{dw}}{W_{dw}}\times 100$$

where W_sw_ is the weight of the swollen film and W_dw_ is the weight of the dried film.

#### 4.3.5. Water Vapor Permeability Analysis of Sericin–PVA Films

The water vapor permeability (WVP) of the sericin–PVA film was determined using a slightly modified ASTM E96-97 method [83]. Sericin–PVA films were cut into circular shapes of appropriate size to fit the mouth of a 50 mL wide-mouth cup. A 10 g calcium chloride anhydrous powder was placed inside the cup as a desiccant, achieving a relative humidity of 0%. The cup was then covered with the sericin–PVA film and sealed with liquid paraffin. The cup’s weight was recorded before it was placed in the desiccator to ascertain its initial weight and that of the desiccant. Inside the desiccator, a second cup was filled with various saturated salt solutions (potassium chloride and magnesium chloride) in its lower section. The desiccator was incubated in a conditioned oven set at 30 °C. The weight of the wide-mouth cup was recorded at 12 h intervals until the difference in mass reached a stable value of no more than 5%. The tests were conducted in triplicate. The water vapor permeability was calculated using the following equation (Equation (3)):

(3) $$\mathrm{WVP}\;(g\cdot \mathrm{mm})/(m^{2}\cdot h\cdot \mathrm{kPa})=\frac{∆m\times L}{A\times t\times ∆p}$$

where ∆m is the mass (g) difference of the wide-mouth bottle, L is the thickness of the film (mm), A is the exposed surface area of the film (m^2^), t is the reaction time (h), and ∆p(RH_1_-RH_2_) is the vapor pressure difference between the two sides of the film (kPa). The results presented in this study demonstrate average vapor permeability values for each sample.

#### 4.3.6. Degradation Analysis of Sericin–PVA Films

The study examined the degradation of sericin–PVA films by submersing them in three media—ultra-pure water, MES (acidic buffer), and PBS (alkaline buffer)—for a period of four weeks. The pre-weighed dried films in the form of a square disc (1 cm^2^ area × 0.05 mm thickness) with an average weight of 0.005 g were immersed in 9 mL of PBS, MES, and distilled water along with 1 mL of 1% sodium azide solution and incubated at 37 °C. After the immersion, the films were removed, washed in distilled water, and dried at 40 °C in an oven for 2 h, and then kept in desiccators. The weight loss of the films was determined at seven-day intervals using an analytical balance. The sericin–PVA films were accurately weighed, and the percentage weight loss for each sample was measured according to the equation (Equation (4)) given below:

(4) $$\mathrm{Percentage}\mathrm{Weight}\mathrm{loss}\;(\%)=\frac{W_{d}-W_{i}}{W_{d}}\times \mathrm{100}\;\%$$

where *W_d_* is the dry weight of sericin–PVA films before immersion and *W_i_* is the weight after immersion in the solution. The experiments were performed in triplicate for each sample, and data were presented as mean ± SD.

#### 4.3.7. Mechanical Properties Analysis of Sericin–PVA Films

The mechanical properties of sericin–PVA films were determined utilizing measurements for tensile strength, elongation, and break strength. The tests were conducted using the Instron Materials Testing System (Instron Corporation, Canton, MA, USA). Uniform film samples measuring 2 mm × 1.5 mm (l × w) strips were prepared from the sericin–PVA films. The sericin strips had a thickness measuring between 0.054 and 0.066 mm and a cross-section of approximately 0.34 mm. Sericin film strips were individually mounted in the pneumatic grips of the testing machine. The pneumatic grips were set at an initial separation of 50 mm, and the crosshead speed was set at 5 mm/min. The measurements of the film strips were performed in triplicate.

#### 4.3.8. Morphological Analysis of Sericin–PVA Films

The sericin–PVA films were cut into pieces and equilibrated at 53% relative humidity before analysis. All the sericin–PVA films were sputter-coated with gold and then examined morphologically using a scanning electron microscope (JEOL Co., Ltd., JSM-IT300HR, Tokyo, Japan) at an accelerating beam voltage of 20 kV.

### 4.4. Antibacterial Efficacy of Sericin–PVA Films

#### 4.4.1. Preparation of Bacterial Inoculum and Sericin–PVA Solvent Extracts

The four bacteria were subcultured in nutrient broth at 37 °C for 24 h to obtain pure isolate inocula of Bacillus subtilis (ATCC 6633), Escherichia coli (ATCC 8739), Staphylococcus aureus (ATCC 25923), and Staphylococcus epidermidis (ATCC 27738). The bacterial strains were offered by the Department of Life and Consumer Sciences at the University of South Africa. The three sericin–PVA solutions were prepared at a concentration of 10 mg/mL and then adjusted to a pH of 3.0 with 0.1 N HCl. L-Ascorbic acid in dimethyl sulfoxide (DMSO) was used as a negative control.

#### 4.4.2. Well Agar Diffusion Assay

The antibacterial susceptibility test method used was the well diffusion technique. The agar medium was prepared by mixing approximately 30 g of nutrient agar powder with 500 mL of deionized water in an Erlenmeyer flask, followed by sterilization. Once sterilized, the agar was cooled to about 45 °C and then poured into Petri dishes to solidify. Pure bacterial isolates, with a concentration of approximately 1 × 10^8^ CFU/mL, were evenly inoculated onto the solidified nutrient agar plates to ensure uniform growth. After drying for 20 min, wells with a diameter of 5 mm were carefully cut into the agar. Then, 30 μL of the rinsed solutions from the sericin–PVA composite were added to each well. The plates were incubated at 37 °C for 24 h. The inhibition zone around each well was measured using a micrometer, with the diameter including the 5 mm well size. Bacterial inhibition was determined by the size of the inhibition zone. All tests were performed in triplicate, and the results were averaged.

## 5. Conclusions

In conclusion, sericin protein can present alternatives for treating burn wounds and ulcers, as it demonstrates excellent biocompatibility, biodegradability, and non-toxicity against cells, as proven by numerous studies. The results of this study have demonstrated the film-forming ability of sericin protein when crosslinked with polyvinyl alcohol, resulting in composite films with enhanced mechanical properties and reduced solubility.

This study should be regarded as an initial step in researching Southern African wild silk sericin protein, which warrants further investigation into its potential applications in wound dressing and drug delivery products.

## Figures and Tables

**Table 1:** Compositions (mol% ± SD) of major amino acids from the three silk sericin extracts.

Sericin Protein (mol% ± SD)			
Amino Acid	*G. postica* (*n* = 6)	*G. rufobrunnea* (*n* = 6)	*Argema mimosae* (*n* = 6)
**Serine ^P^**	31.2 ± 0.11	20.4 ± 0.10	19.6 ± 0.15
**Glycine ^NP^**	21.4 ± 0.26	20.2 ± 0.13	19.8 ± 0.16
**Aspartic acid ^a^**	15.9 ± 0.17	13.4 ± 0.12	14.1 ± 0.16
**Glutamic acid ^b^**	9.82 ± 0.20	9.2 ± 0.05	7.9 ± 0.07
**Alanine ^NP^**	9.5 ± 0.05	11.0 ± 0.10	7.2 ± 0.10

P = polar amino acids; NP = non-polar amino acids; a = aspartic acid is a combination of aspartic acid and asparagine; b = glutamic acid is a combination of glutamic acid and glutamine. *n* = 6 number of measurements.

**Table 2:** Crystallinity percentage of sericin–PVA films.

Sericin Films	Crystallinity Percentage (%Cr)
**GP-SPF**	55.9
**GR-SPF**	17.7
**S** **at** **-SPF**	66.4
**Pure PVA**	30.6

**Table 3:** The moisture vapor transmission rate and thickness of the sericin–PVA films.

Sericin–PVA Film	MVTR (g/m^2^/24 h)		Thickness (mm) (*n* = 4)
	MgCl_2_ (*n* = 4)	KCl (*n* = 4)	
***G. postica*** **(GP-SPF)**	2916 ± 0.02	5162 ± 0.11	0.052 ± 0.11
***Argema mimosae*** **(Sat-SPF)**	1320 ± 0.05	1728 ± 0.35	0.066 ± 0.20
***G. rufobrunnea*** **(GR-SPF)**	1094 ± 0.12	991.2 ± 0.15	0.057 ± 0.13

*n* = number of replicates.

**Table 4:** Mechanical parameters of the three sericin–PVA films.

Films	Tensile Strength [MPa]	Elastic Modulus (Stiffness) [MPa]	Elongation at Break [%]
**GP-SPF**	11.2 ± 0.32	25.0 ± 1.20	107.2 ± 3.11
**GR-SPF**	30.4 ± 1.03	78.8 ± 0.52	29.3 ± 2.32
**Sat-SPF**	15.5 ± 0.25	47.8 ± 0.31	73.0 ± 4.10

GP-SPF: *G. postica*–sericin–PVA film; GR-SPF: *G. rufobrunnea*–sericin–PVA film; Sat-SPF: *Saturniidae*–sericin–PVA film.

**Table 5:** Inhibitory effect of the three sericin species on different strains of bacteria.

Bacteria	Sericin–PVA Composite Films		
	GP-SPF	GR-SPF	Sat-SPF
***S. aureus***	18.90 ± 0.30	14.95 ± 0.09	16.75 ± 0.09
***S. epidermis***	19.63 ± 0.05	16.87 ± 0.2	17.25 ± 0.22
***Bacillus subtilis***	18.45 ± 0.05	15.65 ± 0.1	16.60 ± 0.2
***E. coli***	10.20 ± 0.30	7.70 ± 0.1	8.13 ± 0.01

Diameter of inhibition zone in mm (mean ± SD) (*n* = 4).

## Data Availability

The data presented in this study are available on request from the corresponding author.

## Abbreviations

GP-SPF	*Gonometa postica* sericin–PVA film
GR_SPF	*Gonometa rufobrunnea* sericin–PVA film
Sat-SPF	*Saturniidae* sericin–PVA film
*GA*	Glutaraldehyde
PVA	Poly (vinyl alcohol)
XRD	X-ray diffraction spectroscopy
MVTR	Moisture vapor transmission rate
WVP	Water vapor permeability
pI	Isoelectric points
NMF	Natural moisturizing factors
PBS	Phosphate-buffered saline
MES	2-(N-morpholino) ethanesulfonic acid
ATCC	American Type Culture Collection
